# Will personality traits affect the use of e-cigar among college students? A cross-sectional study in Guangdong Province, China

**DOI:** 10.3389/fpubh.2022.1032606

**Published:** 2022-12-16

**Authors:** Jianrong Mai, Lina Lin, Ling Zhou, Qinyi Guan, Wenhui Zhu, Wenzan Zhou

**Affiliations:** School of Nursing, Guangzhou Xinhua University, Guangzhou, China

**Keywords:** electronic cigarette, personality traits, college students, health education, health policy

## Abstract

**Background:**

The prevalence of e-cigar among adolescents and young adults around the world is increasing rapidly, which has a serious impact on the health of young people. This study assessed the prevalence of e-cigar among college students and to explore the relationship between e-cigar use and personality traits.

**Methods:**

This study conducted an electronic questionnaire survey on college students who were from three undergraduate universities and three junior colleges in Guangdong Province from January 2022 to March 2022. The survey was conducted by stratified cluster sampling, and the respondents were 1362. Statistical descriptions are used to describe the demographic characteristics and personality traits of participants. Mann-Whitney U tests, and Chi-square tests were used to compare the differences between current e-cigar users and non e-cigar users. Two-step hierarchical Logistic regression was used to predict the associated factors with e-cigar use.

**Results:**

The prevalence of current e-cigar users was 5.1%. Agreeableness showed statistically significant higher in non-users (*Z* = 2.585, *P* < 0.01). Moreover, gender (*AOR* = 0.312, *95%CI*: 0.174–0.562), the relationship with mother (*AOR* = 5.887, *95%CI*: 1.460–23.748), friends who use e-cigar (*AOR* = 3.808, *95%CI*: 2.159–6.719), allowance per month (*AOR* = 2.482, *95%CI*: 1.371–4.490), and agreeableness (*AOR* = 0.957, *95%CI*: 0.918–0.997) were related to the use of e-cigar.

**Conclusion:**

The level of agreeableness is associated with the use of e-cigar among college students. All these provided an important theoretical basis for future intervention.

## 1. Introduction

China has the largest number of traditional smokers in the world. With the vigorous promotion of electronic cigarette (e-cigar) manufacturers, the production and consumption of e-cigar has shown a trend of rapid growth in the past few years (1). The number of employees of e-cigar in China exceeded 2 million, with annual sales exceeding 33.7 billion yuan and exports approaching 30 billion yuan in 2018 (2).

Electronic cigarette is an electronic nicotine delivery system, and its impact on health varies. Studies have shown that the use of e-cigar may lead to short-term or long-term health risks (3). The liquid of e-cigar contains glycerol, propylene glycol, natural oil, extract and spices, nicotine and benzoic acid (4). As we know, nicotine can enter the blood circulation through the lungs, stimulating the brain to release dopamine and produce euphoria, which is also the main cause of nicotine addiction (5). Moreover, in order to attract teenagers and young adults to accept e-cigar, manufacturers adopt fashionable designs, good user experiences (for example, reducing irritation to the throat), different tastes (for example, fruit, mint and traditional cigarettes, etc.) and can be used in places where smoking is prohibited (1, 6, 7). These “advantages” that lead to the increasing popularity of e-cigar among college students year by year.

Some studies in China have found that the prevalence rate of e-cigar among college students in Shandong Province was 4.0% in 2015, 7.7% in Shanghai in 2017 and 8.2% in Hangzhou in 2019 (8–10). A study in Pakistan showed that 6.2% of college students had used e-cigar in 2016 (11). 74.9% of Malaysian college students used e-cigarettes in 2016 (12). A cross-sectional survey in New Zealand in 2018 found that 6.1% of college students were current smokers and 40.5% of respondents had used e-cigar (13). The prevalence of e-cigar was 4.0% in the United Arab Emirates in 2020 (14). These studies suggest that the prevalence of e-cigar among college students is getting higher and higher.

Previous studies report that the individual factors, such as age, gender, educational level, allowance per month (Yuan), the relationship with parents, and friends who use e-cigar, may be important factors affecting the use of e-cigar among adolescents. Compared with the elderly, the youth are more likely to accept and use e-cigarettes out of curiosity. The use of e-cigarettes may thought to be cooler and more fashionable, which is one of the important reasons why they use e-cigar (1, 15). Similar to those who use conventional cigarettes, most of the e-cigar users are male (16). A previous study showed that the use of e-cigar was related to educational level (9). The prevalence rate of e-cigar is lower in individuals with higher educational level, which may be related to the awareness of the health impact. Parental rearing style has a direct impact on parent-child relationship and children's stress coping style. Some studies have shown that parental rearing style may affect children's use of tobacco products (17, 18). Peer relationship has been proved to be closely related to healthy behavior. Deviant peer affiliation have been linked to unhealthy behaviors such as smoking, drinking and cyber bullying, which may be a window for college students to be exposed to e-cigar (19).

As early as the 1970s, people began to study the personality traits of smokers. Personality traits are specific and relatively stable characteristics and the main indicators of behavior (20). The Big Five Personality Traits, including neuroticism, conscientiousness, agreeableness, openness and extraversion, are widely used to study the health-related behaviors (21, 22). Neuroticism is thought to be associated with low self-esteem, pessimism and fear. Conscientiousness is related to organized social support. Agreeableness is related to obedience and belief in cooperation. Openness means rich imagination and curiosity about things. Extraversion refers to being good at socializing and having a wide range of interests. Some studies have shown that smoking is associated with high neuroticism, high extraversion, low agreeableness, low conscientiousness and low openness (23, 24).

However, few studies have reported the relationship between e-cigarette smoking status and the Big Five Personality Traits. A previous study on the relationship between three polymorphisms in the dopamine receptor 2 gene and personality traits and anxiety (25). Nevertheless, the relationship between the use of e-cigar and personality traits has not been analyzed from a group point of view. As far as we know, the use of e-cigar is a kind of behavior, which can be intervened by using a variety of theories of health education. Therefore, the purpose of this study is to assess the prevalence of e-cigar among college students, and to address the relationship between the use of e-cigar and personality traits among college students, and to further explore the factors affecting the use of e-cigar.

## 2. Participants and methods

### 2.1. Participants

The purpose of this study is to explore the use of e-cigar and its relationship with personality traits among college students in Guangdong Province, China. Through the questionnaire Star platform (www.wjx.cn), questionnaires were distributed to college students in Guangdong Province. The electronic questionnaires were distributed from January 2022 to March 2022.

According to the calculation, the sample size is 939, based on the fact that the prevalence rate of e-cigar smoker among college students in Shanghai was 4.6%, the margin of error was 3%, the probability of type I error was 5%, the power was 80%, and the rejection rate was 20%. Based on the principle of stratified cluster sampling, a survey was conducted among students from three undergraduate universities and three junior colleges in Guangdong Province. First of all, according to the level of economic development, one undergraduate university and one college from Guangzhou, Foshan and Jieyang are selected respectively. Secondly, a stratified cluster sampling is used and all the students of each grade in each university or college are randomly selected as objects. The inclusion criteria for participants are age ≥16, studying in Guangdong Province, volunteering to participate in the research and completing the consent form. The exclusion criteria are participants with severe mental illness and unwillingness to cooperate. All participants will sign an electronic informed consent form and agree to start the investigation before the investigation. A total of 1,403 questionnaires are collected, and after data cleaning, the valid questionnaires are 1,362. The study is approved by the Biological and Medical Ethics Committee of Guangzhou Xinhua University (2022K002).

### 2.2. Current e-cigar user or non e-cigar user

Participants are required to complete a questionnaire on the use of e-cigar. The e-cigar smoking status is answered by the participants, and they are asked, “Have you used e-cigar in the past 30 days, even 1 or 2 puffs?” Response choices are “Yes” and “No.” If the answer “Yes” is defined as the current e-cigar user, otherwise it is defined as non e-cigar user. The prevalence of e-cigar refers to the percentage of current e-cigar users in the population. The environmental factors of e-cigar smoking include two questions: “Whether you come from a smoke-free family.” and the options are “Yes” and “No.” “Whether any friends use e-cigar.” The option is “0” to “>5.” The answers are changed into dichotomies that is, “0” is “None” and “1” is “Yes.”

### 2.3. Personality traits assessment

The personality traits of participants are measured by Chinese Big Five Personality Inventory Brief Version (CBF-PI-B) (26). In the past few decades, the Big Five Personality Structure model (neuroticism, conscientiousness, agreeableness, openness and extraversion) had been widely studied and proved to have cross-linguistic, cross-calming and cross-cultural stability. And it has been widely accepted by personality psychologists at the dimensional level. CBF-PI-B includes 40 items and 5 dimensions, and each dimension has eight items to measure. Participants respond according to the Likert 6 scale, ranging from 1 (very disagree) to 6 (very agree). In the present study, the Cronbach α coefficient is 0.889, and the Cronbach α coefficients of the five dimensions are between 0.752 and 0.922, indicating a high internal consistency.

### 2.4. Demographic characteristics

The demographic characteristics of the participants include age, gender, educational level (junior college, undergraduate or higher), allowance per month (<900 yuan, ≥900 yuan), the relationship with father (good or bad), and the relationship with mother (good or bad).

### 2.5. Statistical analysis

All statistics analyses are performed using IBM SPSS Statistics (IBM Corp., Armonk, NY, USA) for Windows Version 25.0. The quantitative data in accordance with the normal distribution are represented by mean ± SD, otherwise are expressed by median (Q_1_, Q_3_). Qualitative data are expressed by frequency or percentage (%). χ^2^ tests are used to compare the demographic variables of current users and non-users. Mann-Whitney U tests are used to compared personality traits between current users and non-users. Two-step hierarchical Logistic regression is used to explore the influencing factors of the use of e-cigar. In the first step, hierarchical regression analysis mainly discusses the influence of demographic characteristics on the use of e-cigar. The second step hierarchical regression analysis adds personality variables on the basis of the first step and to explore the influence of personality traits on the use of e-cigar. All reported *P* value (two-sided) are considered statistically significant if *P* < 0.05, with a confidence interval at 95%.

### 2.6. Quality control

Before the formal survey, two rounds of pre-surveys are conducted and the opinions of the feedback are collected and sorted out in time. The questionnaire is revised again after the discussion by the research group. Investigators, who complete the training course and successfully pass the test in December 2021, can participate in the formal investigation. The whole process is guided by the investigator to ensure the quality of the investigation. After the questionnaire is collected, two people will carry out logical check and data screening, and eliminated the questionnaires that are invalid and inconsistent in logical examination.

## 3. Results

### 3.1. Demographic characteristics of the participants

As shown in Table 1, the average age of the participants is (20.0 ± 1.5) years. Of the participants, 51.8% are female and 51.2% are undergraduate or higher. There are 60.5% respondents reporting that their allowance per month ≥900 yuan. 2.8% and 1.1% of the participants report that they have a bad relationship with their parents, respectively. 5.1% of the respondents report that they are using e-cigar, and 42.0% participants have friends who used e-cigar.

**Table 1:** Demographic characteristics of 1,362 participants.

**Characteristics**	**Mean ±SD/*n* (%)**
Age (year)	20.0 ± 1.5
**Gender**	
Male	657 (48.2)
Female	705 (51.8)
**Educational level**	
Junior college	664 (48.8)
Undergraduate or higher	698 (51.2)
**Allowance per month (Yuan)**	
< 900	538 (39.5)
≥900	824 (60.5)
**The relationship with father**	
Good	1,324 (97.2)
Not good	38 (2.8)
**The relationship with mother**	
Good	1,347 (98.9)
Not good	15 (1.1)
**e-Cigar smoking status**	
Non-users	1,293 (94.9)
Current users	69 (5.1)
**Friends' e-cigar users**	
No	790 (58.0)
Yes	572 (42.0)
**Non-smoking family**	
No	874 (64.2)
Yes	488 (35.8)

### 3.2. Differences between non-users and current users by demographic variables

5.1% of the participants reported that they are current e-cigar users. As shown in Table 2, the current e-cigar users have significantly higher proportion of male relative to female (χ^2^= 15.101, *P* < 0.001). Students whose allowance per month larger than 900 Yuan are more likely to use e-cigar (χ^2^= 5.472, *P* = 0.019). There is a statistically significant difference in current users who report that they have a bad relationship with their father (χ^2^= 14.497, *P* = 0.001). Similar to the results of self-report relationship with father, this phenomenon also occurs to students who have a bad relationship with their mother (χ^2^= 25.198, *P* < 0.001). Students who have friends using e-cigar are more likely to become current users (χ^2^= 30.393, *P* < 0.001). However, there are no statistical difference between non-users and current users in terms of age, educational level and whether they come from non-smoking family.

**Table 2:** Comparing the demographic characteristics between non-users and current users.

**Variables**	**Non-users (%)**	**Current users (%)**	***χ^2^/Z***	***P***
Age	20.0 (19.0, 21.00)	20.0 (19.0, 22.00)	0.245	0.807
Gender			15.101	< 0.001
Male	608 (92.5)	49 (7.5)		
Female	685 (97.2)	20 (2.8)		
Educational level			0.690	0.406
Junior college	627 (94.4)	37 (5.6)		
Undergraduate or higher	666 (95.4)	32 (4.6)		
Allowance per month (Yuan)			5.472	0.019
< 900	520 (96.7)	18 (3.3)		
≥900	773 (93.8)	51 (6.2)		
The relationship with father			14.497	< 0.001
Good	1,262 (95.3)	62 (4.7)		
Not good	31 (81.6)	7 (18.4)		
The relationship with mother			25.198	< 0.001
Good	1,283 (95.2)	64 (4.8)		
Not good	10 (66.7)	5 (33.3)		
Friends' e-cigar users			30.393	< 0.001
No	772 (97.7)	18 (2.3)		
Yes	521 (91.1)	51 (8.9)		
Non-smoking family			0.197	0.657
No	828 (94.7)	46 (5.3)		
Yes	465 (95.3)	23 (4.7)		

### 3.3. Personality traits between non-users and current users

As shown in Table 3, respondents who do not use e-cigar, have higher level of conscientiousness and agreeableness, lower level of neuroticism and extraversion. Among the current users, the score of openness is the highest, and follow by conscientiousness, agreeableness, and extraversion, while neuroticism scored the lowest. According to the results of Mann-Whitney U tests, agreeableness shows statistically significant higher in non-users (*Z* = 2.585, *P* < 0.01). There are no significant differences in total score of personality traits and other dimensions between non-users and current users.

**Table 3:** Comparison of the personality traits between non-users and current users.

**Dimension**	**Non-users [M, (Q_1_, Q_3_)]**	**Current users [M, (Q_1_, Q_3_)]**	***Z***
Neuroticism	20.0 (13.0, 28.0)	25.0 (13.0, 31.0)	1.597
Conscientiousness	32.0 (26.0, 38.0)	31.0 (25.0, 40.0)	0.100
Agreeableness	32.0 (28.0, 38.0)	31.0 (27.0, 33.5)	2.585^*^
Openness	31.0 (25.0, 38.0)	34.0 (24.5, 40.5)	1.744
Extraversion	28.0 (24.0, 33.0)	30.0 (24.08, 34.5)	1.482
Total score	146.0 (131.0, 162.0)	153.0 (136.0, 169.5)	1.428

* *P* < 0.01.

### 3.4. Determinants of the use of e-cigar among college students

As shown in Table 4, two-step hierarchical Logistic regression analysis are used to determine the predictors that associated with the use of e-cigar. The purpose of using two-step hierarchical regression analysis is to study the unique influence of personality traits on the use of e-cigar after controlling demographic variables. Therefore, demographic variables such as gender, the relationship with father or mother (good or not good), and friends' e-cigar users (no or yes), allowance per month (<900 or ≥900 Yuan) enter the first step of hierarchical Logistic regression analysis (Model 1). The result shows that the use of e-cigar is influenced by gender, the relationship with mother, friends' e-cigar users, and allowance per month (*P* < 0.01).

**Table 4:** Factors associated with the use of e-cigar among college students.

**Variables**	**Model 1**		**Model 2**	
	***AOR^#^***	***95%CI***	***AOR^#^***	***95%CI***
Age	1.032	0.869–1.227	1.032	0.864–1.231
Gender (Ref. = Male)	0.271^**^	0.154–0.477	0.312^**^	0.174–0.562
The relationship with father (Ref. = Good)	1.646	0.552–4.911	1.256	0.403–3.916
The relationship with mother (Ref. = Good)	6.824^**^	1.740–26.764	5.887^*^	1.460–23.748
Friends' e-cigar users (Ref. = No)	3.895^**^	2.219–6.839	3.808^**^	2.159–6.719
Allowance per month (Ref. < 900 Yuan)	2.312^**^	1.289–4.148	2.482^**^	1.371–4.490
Neuroticism			1.021	0.995–1.048
Conscientiousness			1.013	0.975–1.052
Agreeableness			0.957^*^	0.918–0.997
Openness			1.004	0.967–1.041
Extraversion			1.013	0.971–1.057
Constant	0.003^**^	–	0.003^**^	–

# AOR, adjusted odds ratio.

** *P* < 0.01;

* *P* < 0.05.

Model 2, the second step of hierarchical Logistic regression analysis, includes demographic variables and personality traits such as neuroticism, conscientiousness, agreeableness, openness and extraversion. The result shows that the aforementioned variables in Model 1 retains a significant effect (*P* < 0.05), and agreeableness is related to the use of e-cigar (*AOR* = 0.957, *P* < 0.05). Adjusted odds ratio (*AOR*) for each predictor at two steps of the analysis are presented in Table 4.

## 4. Discussion

To our best knowledge, this study is the first in China to explore the relationship between the use of e-cigar and personality traits among college students. Based on the theoretical study of Big Five Personality Traits, our study shows that there is significant difference between current users and non-users in the dimension of agreeableness, but there are no significant differences in neuroticism, conscientiousness, openness and extraversion. The results of two-step hierarchical Logistic regression analysis show that age, gender, the relationship with mother, friends e-cigar users and agreeableness are associated with the use e-cigar.

The present study shows that the prevalence of current e-cigar users is 5.1%, which is closed to other studies in China, but still at a low level comparing with the United States and New Zealand (10, 27). However, people in China know less about e-cigar. With the popularity of electronic products and the rapid development of domestic e-commerce platform, online shopping has become one of the main shopping channels for college students in China. Chen et al. (1) conducted a survey on Tmall, the largest e-commerce platform in China, and found that there was misleading information in online sales of e-cigar, such as low cost, healthier than traditional cigarettes, no addictive.

This study also shows that the level of agreeableness is higher in non-users than that of current users, which is consistent with Buczkowski et al. (28). This may be related to the fact that individuals with high level of agreeableness are more submissive and altruistic. According to the definition of agreeableness, it is a tendency to sympathize and cooperate, including altruism, trust and other prosocial behaviors (25). People with high level of agreeableness adopt problem-focused coping strategies, including seeking social support, passive endurance, and avoiding conflicts (29).

Although the four dimensions of personality traits, such as neuroticism, conscientiousness, openness and extraversion are not statistically significant in this study, they are proved to be different in other studies on tobacco products and personality. A previous study had shown that current e-cigarette users had higher level of neuroticism than non-users (25). Neuroticism is mainly related to negative emotions such as fear and anxiety. Studies have shown that individuals with high neuroticism are more likely to have an unhealthy lifestyle and have been linked to starting smoking (28, 30, 31). However, Gareth et al. (32) mentions the concept of “healthy neuroticism,” which mean that higher neuroticism and more socioeconomic resources could lead to healthy behavior, such as seeking advice, requiring testing of screening programs and closer monitoring of life style.

In the Big Five Personality Traits model, conscientiousness is considered to be the most closely related to substance use (33). A meta-analysis of 194 studies confirmed that conscientiousness was negatively correlated with smoking, alcohol and drug abuse (34). Kubicka et al. (35) reported that low levels of conscientiousness in childhood could predict smoking in adulthood in a prospective study involving 24-year follow-up. Additionally, individual with low level of conscientiousness is also closely related to smoking relapse in former smokers. Individuals with high level of conscientiousness have better self-control and long-term planning ability, so their lower smoking behavior may be related to the compliance of healthy lifestyle and the adoption of public health advice (31).

Extraverts are considered to be more sociable. Chen et al. (1) found that e-cigarette sales advertisements in China mentioned that e-cigarette had social benefits such as promoting family harmony and establishing interpersonal relationships. Some studies have shown that smokers had higher extraversion scores than non-smokers (30, 31). On the one hand, high extraversion is related to social ability. Since smoking is usually a social activity, individuals with higher extraversion may start smoking and continue to smoke (31). On the other hand, high extraversion is also associated with sensation of seeking stimulation. Similar to openness, nicotine in e-cigarette solution can promote the release of dopamine and satisfy the sensation of seeking stimulation, which is also an important reason why it is difficult for extraverted people to quit smoking (28, 30).

Openness is a personality trait related to divergent thinking and intelligence (36). Previous study found that the degree of openness mainly depends on the function of dopamine in the prefrontal cortex (36). Nicotine in electronic cigarette solution can stimulate dopamine in prefrontal cortex to increase its activity and make users feel happy, so it is considered to be the reason for individuals to start smoking and continue to smoke (37). Similar results have been found in traditional cigarette and personality studies, where individuals with high levels of openness are significantly associated with an increased risk of trying to use cigarettes and lifetime smoking. Previous studies have found that 20.7% of advertisements mention nicotine salt and vaguely claim that the product has a low nicotine content and misled consumers (1). In addition, Coleman et al. (38) believes that openness is similar to susceptibility, suggesting a lack of clear commitment to the use of tobacco products. Margolis et al. (39) found that teenagers had a low perception of the harm of e-cigar, which made them have a strong curiosity and openness to use of e-cigarettes. Therefore, public health educators need to provide college students with adequate and accurate health education about the effects of e-cigar on health, so as to reduce their curiosity and open attempts to e-cigar. In addition, government need to regulate e-cigarette advertisements to prevent misinformation about e-cigarettes to adolescents and young adults.

Moreover, age is thought to be one of the risk factors for the use of e-cigar. A previous study showed that e-cigar was more easily accepted by adolescents and young adults because it looked fashionable (15). Compared with traditional cigarettes, e-cigar have more flavors, such as fruit, candy, mint, etc. and are more popular with adolescents and young adults. In addition, e-cigar in China is mainly sold to adolescents and young adults, so the online publicity platform has become the main point of sale. However, many e-cigar advertisements gave the wrong message, such as no nicotine, no addiction, can effectively help quit smoking, and arouse strong curiosity among college students (1).

There are also gender differences in e-cigarette users. Overall, the prevalence rate of e-cigar in female are lower than that in male. A study in Japan showed that female smokers had higher extraversion and lower agreeableness than female non-smokers, while male smokers showed higher extraversion and lower openness than male non-smokers (30). Zhao et al. (40) found that female e-cigar users generally used e-cigar for social purpose, which is consistent with the higher extraversion mentioned above. Compared with male e-cigar users, female users will pay more attention to the appearance and design of e-cigar. However, the motivations for male to use e-cigar included use convenient, can help quit smoking, have a similar taste to traditional cigarettes, less harm to health, safety, fashion, can be used in non-smoking places, etc. (40).

In present study, the relationship with mother is considered to be another risk factor for e-cigar use. Students who have a bad relationship with mother are more likely to use e-cigar than those who have a good relationship with their mother. Studies have shown that there is a relationship between parental rearing styles and children's stress coping styles (17, 18). In traditional Chinese families, mother plays a major role in family care and upbringing of children. Mother can teach their children how to cope with stress in their daily life. Children who have a good relationship with their mother may get more attention and care, and their neuroticism (related to negative emotions such as anxiety and fear) have a lower score. Tobacco products have been shown to be associated with stress coping. People with high levels of neuroticism are more likely to use tobacco products to cope with stress (30).

Friends who use e-cigarettes are also closely related to the use of e-cigar, which is consistent with the results of other studies (10, 27). Deviant peer affiliation may be linked to unhealthy behaviors such as smoking, drinking and cyber bullying, which may be a window for college students to be exposed to e-cigarettes (19). When college students face with stressful life events, the cigarette delivery behavior of deviant peers is more likely to lead to the use of e-cigarettes. In addition, college students, who are exposing to the e-cigarette environment, are likely to use by the smoke of e-cigar, curiosity and taste. Therefore, friends who use e-cigar are closely related to the use of e-cigar.

Government should issue corresponding laws to restrict the sales approach of e-cigarettes, especially through the Internet. In addition, relevant laws and regulations should be formulated to prohibit the use of e-cigarettes in public places, so as to provide a good smoke-free support environment for teenagers and college students. Educational institutions should pay attention to tobacco health education activities and carry out courses related to the hazards of e-cigarettes to help teenagers realize the health hazards of e-cigarettes and reduce the possibility of using e-cigarettes. Teachers and staff should also avoid the use of e-cigarettes in schools and set an example for the establishment of a smoke-free campus (41). Family support is also an effective way to reduce the use of e-cigarettes. Parents can reduce the use of e-cigarettes among teenagers through education and supervision. The establishment of smoke-free families helps to protect teenagers from the effects of secondhand smoke, thereby reducing the possibility of using e-cigarettes (42).

The present study has several strengths. To our knowledge, this is the first study to explore the relationship between the use of e-cigar and personality traits among college students in Guangdong Province, China. We find evidence that personality trait, especially agreeableness, is associated with the use of e-cigar. As a protective factor, individuals with higher agreeableness are less likely to use e-cigar. In addition, other personality traits such as neuroticism, conscientiousness, openness, and extraversion should not be ignored, although they are not statistically significant in this study.

There are also some limitations in this study. First of all, the subjects of our study are selected only from three undergraduate universities and three junior colleges in Guangdong Province, and the representativeness may be limited. In the future, our conclusion will be verified by increasing the sample size. Secondly, this is a cross-sectional study, and it is difficult to draw causal conclusions, but it can still provide clues for future longitudinal research. Finally, our study is a self-reported questionnaire, which may have recall bias or information bias. For example, some college students may not want to report the actual status of e-cigar use, so the actual prevalence rate of e-cigar use may be higher. Despite these limitations, our research confirms that there is a relationship between e-cigarette use and personality traits. All these provide an important theoretical basis for future intervention.

## 5. Conclusions

Overall, this study found that the prevalence rate of e-cigar among college students in Guangdong Province, China was 5.1%. In addition, it is confirmed that personality trait, especially agreeableness, is related to the use of e-cigar, but the effects of other personality traits such as neuroticism, conscientiousness, openness and extraversion on e-cigar use can't be ignored. In addition, notable predictors included gender, the relationship with mother and friends who used e-cigar, allowance per month are the factors associated with the use of e-cigar. These phenomena also sound the alarm for Chinese tobacco control departments and government departments. On the one hand, college students' awareness of the harm of e-cigarettes should be raised, on the other hand, relevant policies should be put forward to control the sales and use restrictions of e-cigar.

## Data availability statement

The original contributions presented in the study are included in the article/supplementary material, further inquiries can be directed to the corresponding author.

## Ethics statement

The studies involving human participants were reviewed and approved by Biological and Medical Ethics Committee Guangzhou Xinhua University. The patients/participants provided their written informed consent to participate in this study.

## Author contributions

JM contributed to conceiving and designing the study, wrote the manuscript, and edited the final version. LL and LZ contributed to discussing and analyzing the data. QG, WZhu, and WZhou contributed to data collecting and coding. All authors contributed to the article and approved the submitted version.
